# Supplementary material for: A New Phosphorous/Nitrogen-Containing Flame-Retardant Film with High Adhesion for Jute Fiber Composites
Source: Polymers (Basel). 2023 Apr 18;15(8):1920. doi: 10.3390/polym15081920 (PMC10144265; doi:10.3390/polym15081920)
Supplement: Supplementary file 1 [file polymers-15-01920-s001.zip › polymers-2291943-supplementary.pdf]

A New Phosphorous/Nitrogen-Containing Flame-Retardant Film with High Adhesion for Jute Fiber Composites

Yanli Dou, Zheng Zhong, Jiaming Huang, Aixun Ju, Weiguo Yao, Chunling Zhang and Dongbo Guan *

The Ministry of Education Key Laboratory of Automotive Material, College of Materials Science and Engineering, Jilin University, Changchun 130025, China
* Correspondence: guandb@jlu.edu.cn

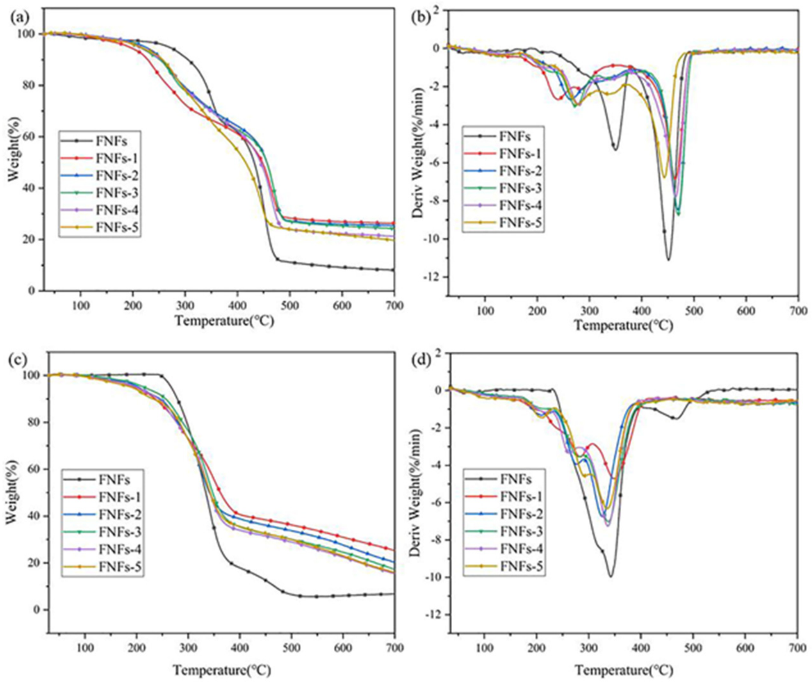

Figure.S1 TGA (a) and DTG (b) curves for FNFs and coated FNFs under N_2 atmosphere, TGA (c) and DTG (d) curves for samples in air

Table S1 Thermal properties of the FNFs and coated FNFs

**Samples**	$T_{0.05}$ （°C）		T_{max} （°C）		CR at 700℃ （wt.%）	
	N_2	Air	N_2	Air	N_2	Air
FNFs	268.0	267.0	455.2	344.7	7.7	6.7
FNFs-1	225.1	196.8	465.0	346.8	25.9	25.3
FNFs-2	229.3	203.3	469.1	326.8	25.2	20.2
FNFs-3	214.2	214.1	471.6	337.5	23.9	17.2
FNFs-4	204.6	199.2	466.5	337.9	20.8	15.4
FNFs-5	192.0	190.1	447.7	331.7	18.7	16.0
